# A Point-of-Care Noninvasive Technique for Surrogate ICP Waveforms Application in Neurocritical Care

**DOI:** 10.1007/s12028-023-01786-2

**Published:** 2023-07-12

**Authors:** Sérgio Brasil, Daniel A. Godoy, Gregory W. J. Hawryluk

**Affiliations:** 1 Division of Neurosurgery, Department of Neurology, School of Medicine, University of São Paulo, São Paulo, Brazil; 2 Neurointensive Care Unit, Sanatório Pasteur, Catamarca, Argentina; 3 Department of Neurosurgery, Cleveland Clinic and Akron General Hospital, Fairlawn, OH USA

## The Historic Narrative

In 2006, Sérgio Mascarenhas, a retired physics professor from the University of São Paulo (Brazil), underwent a ventriculo-peritoneal shunt procedure for normal pressure hydrocephalus (NPH) after a year of unsuccessful treatment for Parkinson’s disease. He became intrigued by the difficulties involved in diagnosing NPH and the lack of noninvasive techniques for assessing intracranial pressure (ICP). Such curiosity led to a significant breakthrough discovery.

Professor Mascarenhas questioned the validity of the Monro-Kellie doctrine, which stated that the skull is nonexpandable [1, 2], despite the fact that even diamonds expand when subjected to pressure. This inquiry drove him to make the first discovery of a linear correlation between cranial microexpansion and changes in ICP [3].

Gustavo Frigieri, a former mentee of Professor Mascarenhas, played a leading role in the studies that translated skull microexpansions into the acquisition of ICP pulse waveform (ICPW). These studies demonstrated a high degree of correlation with the ICPW obtained using intracranial catheters in experimental research [4, 5]. Building on this foundation, engineering research and development efforts resulted in the development of a fully noninvasive wearable device [6], named Brain4Care (B4C).

The B4C system has unveiled a new vital sign with numerous potential medical applications. Professor Mascarenhas passed away in 2021; however, his legacy lives on through his work presented in this article.

## Clinical Justification for ICP Waveform Application

The analysis of ICPW has significantly contributed to the understanding of intracranial hemodynamics. ICPW is generated by the transmission of arterial pressure at the level of the choroid plexus to the cerebrospinal fluid and brain parenchyma [7–9]. The standard ICPW consists of three components: P1, P2, and P3. P1, known as the “percussion wave,” represents arterial pulsation. P2, often referred to as the “tidal wave,” is believed to be associated with intracranial compliance (ICC). Lastly, the P3 component occurs after the closure of the aortic valve, resulting in a temporary halt in blood flow and a drop in ICP. This produces the dicrotic notch “N” and is followed by venous outflow [9–11]. It is worth noting that the arterial blood pressure (ABP) waveform differs from the ICPW due to the brain tissue’s “resistor” function and venous pressure [1]. Consequently, ICPW can reflect the dynamic balance of ICC in relation to continuous arterial pulsations, venous outflow, and cerebrospinal fluid movements [12, 13], providing insights into Δvolume/Δpressure changes [14–17].

**Fig. 1:**
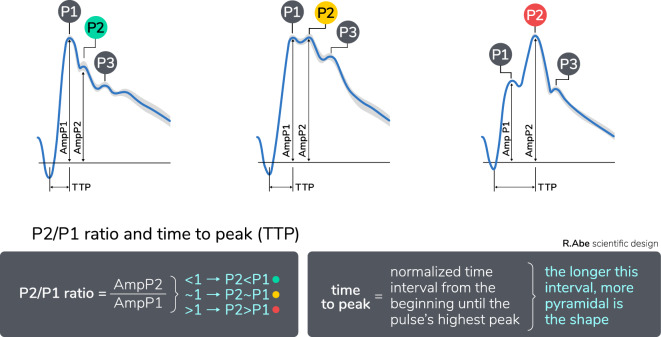
From left to right the ICPW evolution according to ICP elevation. P2:P1 ratio and time-to-peak parameters are automatically derived from noninvasive waveform analysis. ICPW, intracranial pressure waveform, TTP, time-to-peak

As intracranial volume increases, ICPW changes following ICP elevation, indicating the progressive exhaustion of compensatory mechanisms with the resultant increase of the P2 component relative to P1 (Fig. 1) [8, 18–20]. These parameters, specifically the altered P2:P1 ratio, have been recognized as markers of impairment in ICC during studies involving intracranial volume and pressure. They serve as valuable indicators of personalized ICP decompensation [13].

Nucci et al. [8], using an artificial neural network, mapped ICP pulse morphology according to pathological states and observed a progressive increase in the amplitude of P2 relative to P1 as indicative of intracranial hypertension (IH). Furthermore, IH severity is higher if P3 amplitude also overcomes P1 amplitude [8]. Considering this evidence, Brasil et al. [20] applied a controlled ICP elevation in 57 neurocritical patients to test the correlation between ICP measured invasively with P2:P1 ratio and the time-to-peak (TTP) interval obtained with the B4C system (B4C; Braincare Corp, São Carlos, Brazil) (Fig. 1). This study revealed significant elevations in the P2:P1 ratio following ICP increase (*p* = 0.01) [20]. Other researchers have also explored ICPW and have developed moving-average indexes that correlate the ICP pulse amplitude with either ABP [21] or directly measured ICP values [22]. These studies used dedicated software and hardware to analyze and interpret the ICPW data.

## Noninvasive ICP Monitoring

Currently, the most used noninvasive techniques for bedside neuromonitoring have shown varying degrees of performance in assessing ICP, particularly in determining the presence or absence of IH [23]. These techniques are based on ultrasound, such as transcranial Doppler and measurement of optic nerve sheath diameter, as well as pupillometry [24–26]. However, until recently, there was no bedside method available specifically designed to provide a surrogate ICPW and assess its physiological information.

To date, there have been few approaches attempting to obtain reliable noninvasive ICPW, with varying degrees of success. One such approach was conducted by Evensen et al. [27], who used transfer function analysis to estimate ICPW from brachial ABP waveforms. However, they found only a fair estimation of the ICP waveform in a third of their sample and did not recommend its use for clinical management [27]. More recently, Dixon et al. [28] developed an algorithm using photoplethysmography to assess the correlation between morphological features of the photoplethysmography waveform and ICP levels. This study involved 24 simultaneous recordings in 12 patients with ABI without skin or skull injuries. The authors observed a significant correlation between the morphology of the brain pulse wave and ICP levels (*R*^2^ = 0.66, *p* = 0.001). However, further validation studies with larger and preferably blinded samples are needed to confirm these findings [28].

**Fig. 2:**
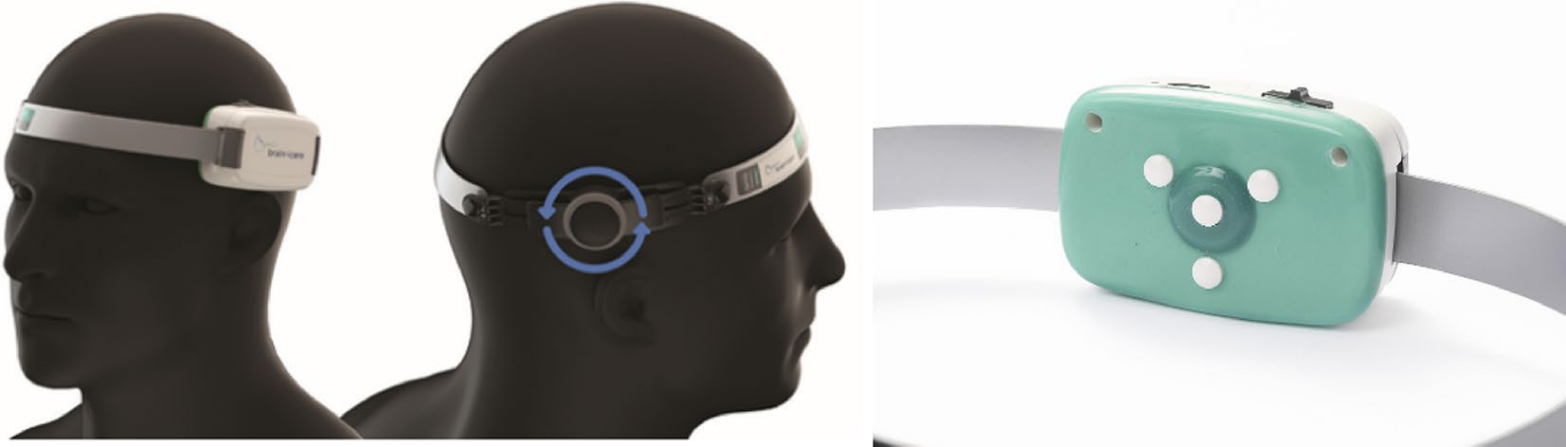
B4C system adaptation to head surface (left) and sensor’s internal surface (right). B4C, Brain4Care

While invasive techniques can display intracranial ICPW on dedicated monitors, they do not typically provide automated waveform analysis or real-time derived parameters. As a result, the interpretation of pulse morphology relies on the expertise of the practitioner. Additionally, it is not uncommon to observe poor resolution waves on monitor screens, making it difficult to identify the peaks of the ICPW at a glance. In contrast, the B4C system has shown satisfactory results in clinical settings. This wearable system, cleared by the Food and Drug Administration (Food and Drug Administration number K201989), generates a surrogate ICPW and performs automated analysis [29, 30]. The engineering principle behind the B4C system involves placing an extremely sensitive pin in contact with the skin over the skull [6]. This pin detects micrometric pulsatile bone expansion. The optimal sensor positioning is approximately 3 cm above the anterior third of the orbitomeatal line and is supported by a headband with a gentle tightening mechanism [29] (Fig. 2). The B4C system generates a real-time report that includes parameters such as the P2:P1 ratio and TTP [29] (as described earlier in the “ICP waveform” section).

In patients with ABI, the B4C waveform has demonstrated high agreement and Pearson correlation with invasive ICP waveform measurements. The P2:P1 ratio showed a correlation coefficient (*r*) of 0.72, whereas the TTP showed a correlation coefficient of 0.85. The B4C waveform also exhibited strong predictive capability for detecting ICP values above 20 mm Hg (*p* < 0.001), with an area under the curve of 0.9. However, it is worth noting that the predictive power for IH decreased when patients underwent neurosurgical procedures [29]. In another study specifically focusing on patients with ABI with undamaged skulls, the B4C waveform demonstrated a 100% negative predictive value (NPV) for the P2:P1 ratio and a 91.7% NPV for TTP, using cutoff values of 1.06 and 0.2, respectively. These values indicate that the B4C waveform can effectively rule out the presence of IH in these patients [30]. Table 1 summarizes the most relevant studies for validation of this technique to date.

**Table 1:** Main findings of the studies for validation of the cranial pulsation sensor (B4C)

Study	Sample	Population	Main results	Conclusions
Brasil et al. [29] J Person Med	41	TBI, SAH, and IS	Patients with preserved cranial integrity exhibited the best linear correlations for both P2:P1 ratio and TTP (*r* = 0.72 and 0.85, respectively), with P2:P1 ratio > 1.2 an AUROC 0.9 (*p* = 0.001) as predictor of ICP > 20 mm Hg. These same parameters from invasive waveforms showed similar results	The parameters provided by the B4C device are comparable to the invasive intracranial pressure pulse morphology. Invasive and noninvasive pulse morphologies were better correlated among themselves than with the ICP values. The system is able to indicate IH especially among undamaged skull patients
De Moraes et al. [30] Neurocrit Care	18	SAH and ICH	nICPW P2:P1 ratio had 100% NPV and sensitivity for the cutoff 1.06. The areas under the curve to estimate intracranial hypertension were 0.786 (95% CI 0.72–0.93) for the P2:P1 ratio and 0.694 (95% CI 0.60–0.74) for TTP	Our study showed a high correlation and agreement in the analysis of wave morphology between the new B4C method when compared with ICP monitoring, especially the P2:P1 ratio
Brasil et al. [31] J Clin Monit Comp	72	TBI, SAH, and IS	There was a significant correlation between ICP and P2:P1 (*r* = 0.49, *p* < 0.001 Fig. 1). P2:P1 was significantly higher in patients with IH (Fig. 2); P2:P1 had an AUROC to predict IH of 0.88 (95% CI 0.78–0.98), whereas the P2:P1 cutoff of > 1.2 showed a sensitivity of 85% (95% CI 58–97%) and a specificity of 77% (95% CI 64–85%). ICP and BCI were significantly higher for ED group compared with groups SB and MV. The AUROC of P2:P1 to predict early death was 0.71 (95% CI 0.53–0.87)	The B4C system can analyze biometric parameters extracted from the ICPW parameters obtained from cyclic spontaneous cranial deformation, which are correlated with ICP. These parameters seem to be adjuvants for intracranial compliance monitoring and may participate on the outcomes of patients with acute brain injury
Hasset et al. [39] Neurocrit Care	24	SAH	The correlation between Pax and nPAx was strong (*r* = 0.70, *p* < 0.0005, 95% CI 0.687 to 0.717). Bland–Altman analysis showed excellent agreement, with a bias of − 0.018 (95% CI − 0.026 to − 0.01) and a localized regression trend line that did not deviate from 0	Pax can be calculated by conventional and noninvasive ICP monitoring in a statistically significant evaluation with strong agreement. It allows the noninvasive assessment of cerebrovascular reactivity
Frigieri et al. [40] Int Care Med	72	TBI, SAH, and IS	ICS score 0 was associated with a NPV of 100%, whereas ICS score 3 held a positive predictive value of 100% (Additional file 2: Table S1) for ICC impairment. No early death patients had an ICS score of 0, whereas none of the survivors had an ICS score of 3	The ICS is a reliable tool for the detection of ICC alterations. Furthermore, it had better overall performance than individual analysis of ICPW peak amplitudes or time intervals for the prediction of the prespecified outcome measures

AUROC, area under the curve, B4C, Brain4Care, BCI, brain compliance index, CI, confidence interval, ED, emergency department, ICH, intracranial hemorrhage, ICP, intracranial pressure, ICPW, intracranial pressure pulse waveform, ICS, intracranial compliance scale, IH, intracranial hypertension, IS, ischemic stroke, MV, mechanical ventilation, nICPW, noninvasive intracranial pressure waveform, NPV, negative predictive value, Pax, pulse-amplitude index, SAH, subarachnoid hemorrhage, SB, spontaneous breathing, TBI, traumatic brain injury

The B4C system has been suggested as a complementary tool for monitoring patients alongside ICP measurement. It has shown that waveform parameters can improve the ability to determine an individualized threshold for harmful ICP levels [31]. In the context of hydrocephalus, for instance, the system has been used to evaluate children with shunt malfunction [32, 33]. While further studies are necessary, the system may be considered for the evaluation of nonprimary neurological disorders such as robotic prostatic surgery [34], acute respiratory distress syndrome and end-stage renal disease [35–37], as well as a screening method for brain death assessment [38].

In addition to studies evaluating the correlation between B4C waveforms and invasive ICP values and waveforms, another parameter that has been studied is the noninvasive pulse amplitude index of cerebrovascular reactivity. This index has been validated in patients who underwent concurrent invasive ICP monitoring, offering a noninvasive method to assess cerebral autoregulation based on ICPW measurements [39]. This provides a valuable tool for evaluating the dynamic regulation of cerebral blood flow and its relationship to ICP without the need for invasive procedures.

In more recent developments, an artificial intelligence algorithm has been developed using a large data set of B4C data cross correlated with invasive ICP measurements. This algorithm has allowed for the creation of a scale to quantify ICC. According to this scale, a value of zero indicated a 100% NPV, meaning a low likelihood of ICC impairment, whereas a value of three indicated a 100% positive predictive value, indicating a high likelihood of ICC impairment [40]. This scale provides a useful tool for assessing and predicting the presence of ICC impairment based on B4C data.

## The Benefits of Noninvasive ICP Monitoring

The main obvious limitation for ICP monitoring is the need for a neurosurgical procedure, which carries the risks of hemorrhages and infections. Furthermore, the cost associated with invasive monitoring makes it less accessible in resource-constrained regions where cost reduction in medical care is crucial. In this regard, the B4C system offers advantages. Unlike invasive techniques, the B4C system is not disposable and can be used to perform assessments on multiple patients, making it potentially more cost-effective. This feature can contribute to reducing the overall expenses associated with ICP monitoring and make it more accessible in regions where resources are limited. By providing a noninvasive alternative, the B4C system has the potential to address the limitations of traditional invasive methods and make ICP monitoring more feasible in various health care settings.

Another noteworthy aspect of the B4C approach is its ability to explore ICC in various clinical situations that pose an imminent risk of IH but are difficult to identify through imaging or lumbar puncture procedures alone. Conditions such as idiopathic IH, NPH, and ventriculo-peritoneal shunt malfunction can benefit from the assessment of ICC using the B4C system. Furthermore, the portability and ease of use of the B4C system make it potentially suitable for prehospital settings and emergency departments for the triage of neurological deterioration.

Ultimately, noninvasive monitoring would be indicated for those with nonprimary neurological disorders but who are at risk of cerebrovascular derangements and consequently ICC impairment, such as in sepsis, severe pulmonary diseases, extracorporeal circulation, and liver failures [31, 41] and when an invasive procedure is contraindicated (i.e., coagulopathies).

## Roads for Improvement

Currently, B4C has two major limitations. Firstly, the device is sensitive to patient movements, which may generate artifacts. However, these artifacts are reduced by a cloud-based algorithm processing tool. In this tool, the waveforms are analyzed, the pulses are identified, and a high filter for signal-to-noise ratio is applied. The parameters are calculated in real time and reported after this processing. Secondly, there is operator dependence on recognizing optimal waveform acquisition, as inadequate positioning may alter the results. It is essential for the responsible physician and/or nurse to remain attentive to sensor displacements during the monitoring session. Therefore, it is advised to conduct short serial monitoring sessions of approximately 15 min each, based on clinical judgment and need. In the near future, engineering improvements may address these issues and provide a solution.

Regarding the analytics data provided by the platform, there is a lack of normative data for the P2:P1 ratio and TTP biometrics from healthy individuals of different age groups and sex. The parameters obtained to date have primarily been derived from severely ill patients. However, there is an ongoing study (available at https://ensaiosclinicos.gov.br/rg/RBR-9nv2h42) that aims to address this need. Moreover, in its current hardware version, the device may not be suitable for acquiring proper data from patients with a small skull circumference (approximately under 34 cm), such as pediatric patients under 1 year old or those with congenital malformations.

To date, studies have provided insights into the clinical applicability of this method and validated its information by correlating it with invasive ICP probes. The next steps in research may focus on examining the correspondence of this technique with other noninvasive methods such as pupillometry, transcranial Doppler, and optic nerve sheath ultrasound measurements [42]. Additionally, it is important to assess the value of this information in patient management and gather prognostic data. Furthermore, it should be noted that the B4C system is currently unable to provide an estimate of the actual ICP absolute value. However, artificial intelligence and machine learning techniques are being applied, and preliminary results are promising (available at https://journals.lww.com/neurosurgery/Abstract/2023/04001/332_Estimation_of_Intracranial_Pressure_Using.156.aspx) [43].

## Conclusions

A reliable, noninvasive assessment of ICC offers several advantages over current invasive techniques for monitoring ICP, apart from the obvious benefit of avoiding skull drilling. Exploring ICP pulse morphology is a promising avenue in this regard, although only a few techniques have shown potential for obtaining accurate noninvasive ICP waveforms to date. The noninvasive system described in this report, which enables micrometric skull expansion detection, appears to be the most viable method for acquiring and analyzing surrogate ICP waveforms. It can serve as a screening tool for neurosurgical procedures and be used in conjunction with invasive techniques or when invasive ICP monitoring is unavailable, not recommended, or contraindicated. The uniqueness and originality of this method have the potential to establish it as a new vital sign that can be universally explored. Further research investigating therapeutic strategies based on the information provided by this technique may help define its role in neurocritical care.
